# Design, synthesis, and SAR study of isopropoxy allylbenzene derivatives as 15-lipoxygenase inhibitors 

**DOI:** 10.22038/ijbms.2020.36793.8763

**Published:** 2020-08

**Authors:** Mina Mousavian, Seyed Jamal Alavi, Raheleh Rahbarian, Majid Rajabian, Hossein M. Orafai, Hamid Sadeghian

**Affiliations:** 1 Department of Biology, Faculty of Science, Payame Noor University, Mashhad, Iran; 2 Department of Laboratory Sciences, School of Paramedical Sciences, Mashhad University of Medical Sciences, Mashhad, Iran; 3 Department of Pharmaceutics, Faculty of Pharmacy, University of Al-Zahraa for Women, Karbala, Ira**q**; 4 Neurogenic Inflammation Research Center, Mashhad University of Medical Sciences, Mashhad, Iran

**Keywords:** 15-lipoxygenase, Allylbenzene, DMAB, Kinetic, MBTH

## Abstract

**Objective(s):**

Allylbenzenes have been recently developed as inhibitors of lipoxygenases. They decrease peroxidation activity via mimicking 1,4-unsaturated bonds of fatty acids by their allyl portion. We designed and synthesized new derivatives of allyl benzenes (**6a-f**) with isopropoxy and amide substituents at ortho and meta positions towards allyl group, respectively. The inhibitory potency of the synthetized allylbenzenes against soybean 15-lipoxygenase (SLO) and subsequently structure-activity relationships was assessed.

**Materials and Methods:**

3-allyl-4-isopropoxybenzenamine (**5**) as starting material was synthesized by coupling of 4-nitropheol with allyl bromide, performing Claisen rearrangement and finally reduction of the nitro moiety. Final products **6a-f **were prepared via amidation of **5** with the desired acyl chloride.

**Results:**

Among the compounds, N-(3-allyl-4-isopropoxyphenyl)adamantan carboxamide (**6f**) potentially showed best inhibition (IC_50 _= 1.35 µM) while **6a** with cyclopropyl carboxamide moiety was the weakest inhibitor and **6e** with phenyl carboxamide moiety showed no effect. Energy minimized 3D structures of the compounds were docked into the active site pocket of SLO. For the aliphatic amides, docking results showed compatibility between inhibitory potency and average Ki of the cluster conformers, in which their allyl moiety oriented towards SLO iron core. For the aliphatic analogs, by enlargement of the amide moiety size the inhibitory potency was increased.

**Conclusion:**

Docking results showed that orientation of the amide and allyl moieties of the inhibitors in the active site pocket is the major factor in inhibitory potency variation. Based on the mentioned orientation, for cycloaliphatic amides, by enlargement of the amide moiety both inhibition potency and calculated binding energy increases.

## Introduction

Lipoxygenases (LOXs) are enzymes that catalyze polyunsaturated fatty acids peroxidation at a specific position of the aliphatic chain. In humans there are six types of LOXs: 5-LOX (arachidonate 5-lipoxygenase), 12-LOX (arachidonate 12-lipoxygenase; platelet lipoxygenase), 12/15-LOX or 15-LOX-1(arachidonate 15-lipoxygenase-I; reticulocyte 15-lipoxygenase) and 15-LOX-2 (arachidonate 15-lipoxygenase-II), 12R-LOX (arachidonate 12R-lipoxygenase), and eLOX-3 (Epidermis-type lipoxygenase). These different LOXs have been named according to the position and stereochemistry of arachidonic acid peroxidation (1). Linoleic acid (LA) is the most specific substrate for 15-LOX-1 and this enzyme metabolizes LA to 13(S)-HPODE (13(S)-Hydroxyperoxy-9Z,11E-Octodecadienoic acid) (2). The peroxidation of arachidonic acid in the presence of 15-LOX-1 mainly produces 15(S)-HPETE and also 12(S)-HPETE (3, 4). This enzyme is well known due to its effective role in many acute and chronic disorders such as inflammation, allergy, and cancers (5, 6). This enzyme also can generate and develop atherosclerosis via oxidizing cholesterol esters of LDL and cell membrane phospholipids (7). Cell based studies have implicated that both Alzheimer’s and Parkinson’s diseases were related to 15-LOX-1 activity in neuronal models of oxidative stress (8, 9).

According to published literature, there is considerable interest in developing of LOX inhibitors for therapeutic purposes. In this study, we evaluated soybean 15-LOX (SLO) inhibitory activities of some new compounds based on the previous studies on allyl benzenes (6, 10, 11). Allyl benzenes are molecules that have been created via single bond junction between the propenyl moiety and phenyl ring; they have been recently introduced as lipoxygenase inhibitors. It has been reported that these compounds decrease lipoxygenase activity probably via mimicking 1,4-unsaturated bonds of linoleic or arachidonic acid by their allyl moiety (10, 12, 13). We designed and synthesized new derivatives of allyl benzenes (**6a-f**) that possess both isopropoxy and allyl moieties at *meta* and *para* positions towards an amide substituent. Inhibitory potency of the synthetic allylbenzenes against SLO (L1 Type; EC 1,13,11,12), structure-activity relationships (SAR), and finally inhibitory mechanism of the most potent inhibitor were evaluated.

## Materials and Methods

***Instruments***

1H NMR (300 MHz) spectra were performed on a Bruker Avance DRX-300 Fourier transformer spectrometer. Chemical shifts are reported as ppm (δ) compared to tetramethylsilane (TMS). The melting points were measured by an Electrothermal type 9100 melting point apparatus. All measurements of UV-visible absorbance were carried out using a Spekol 1500 spectrophotometer and a BioTek ELX800 plate reader. 

***Molecular modeling, docking, and SAR study***

*Structure optimization*

The structures of the desired compounds were drawn in ChemDraw Ultra 8.0. The output files were geometrically minimized under semi-empirical AM1 in HyperChem 7.5 (RMS gradient= 0.1 kcal/mol; algorithm= Polak-Ribiere). The crystal structure of the soybean lipoxygenase-3 (arachidonate 15-lipoxygenase) complexed with 13(S)-hydroproxy-9(Z)-2,11(E)-octadecadienoic acid was retrieved from RCSB protein data bank (PDB entry: 1IK3).

*Molecular docking*

Docking simulation between the 3D structures of SLO and inhibitors was done with AutoDock 4.2 using the Lamarckian genetic algorithm. Torsion angles of ligands, protein hydrogens and bond distances were edited and solvent parameters of the protein were added. Partial atomic charges were then considered for both ligand and protein (Gasteiger for ligand & Kollman for protein). The docking regions were defined by considering the following: Cartesian charts: 18.5, 5.0 and 20.0; grid sizes: 50, 55 and 60 points for X, Y and Z axes, respectively. The docking parameter files were defined for Genetic Algorithm and Local Search Parameters (GALS). “Maximum number of energy evaluations” and “number of generations” were set to 2500,000 and 100, respectively. The docked complexes were clustered with RMSD = 2.0 A^°^. Docking results were imported to Accelrys Ds Visualizer 2.0.1 for further studies.

***Enzyme assays***

*Enzyme inhibition*

LA, enzyme and two assay solutions (A and B) were firstly prepared as follow: 

LA solution: A mixture of LA (5.6 mg), ethanol (0.5 ml) and KOH (100 mM) to a final volume of 5 ml (6).

The enzyme solution was prepared by dissolving 1 mg lyophilized SLO (L1; Type I-B; EC 1, 13, 11, 12; Sigma Co.) in 5 ml phosphate buffer (50 mM, pH 7.0) and then its activity was checked by diene formation method at 234 nm. Finally, its activity was decreased to desired quantity by dilution with the phosphate buffer.

Solution A: DMAB (3-dimethylaminobenzoic acid) 50 mM in phosphate buffer (100 mM, pH 7.0). 

Solution B: A mixture of MBTH (3-methyl-2-benzothiazolonhydrazone) (10 mM, 3 ml) and hemoglobin (5 mg/ml, 3 ml) in 25 ml phosphate buffer (50 mM, pH 5.0). 

Assay procedure: the sample solution in dimethyl sulfoxide (25 µl), enzyme solution (4000 units/ml; 25 µl), and phosphate buffer (pH 7.0, 50 mM; 900 µl) were mixed in a test plate and pre-incubated for 5 min at room temperature. Control test was done using the same volume of dimethyl sulfoxide. After pre-incubation, LA solution (50 µl) was added (starting of the peroxidation reaction) and 10 min later, solution A (270 µl) and subsequently solution B (130 µl) were added to start the color formation. 3 min later, 200 µl of sodium dodecyl sulfate solution (2% W/V) was added to terminate the reaction. The absorbance was recorded at 598 nm. Each experiment was performed in triplicate. The data analysis was done in GraphPad Prism 6.0 (6).

*Inhibitory mechanism*

The experiment was done in Tris buffer (pH 7.2, 50 mM). Five concentrations of LA (6.25, 12.5, 25, 50 and 100 µM) were used. The enzyme activity was measured in the absence (control test) and presence of **6f** as inhibitor (concentrations 125, 100, and 75 µM).

25 µl of the enzyme solution (4000 unit/ml) was added to mixture of buffer (900 µl) and inhibitor (25 µl) and incubated for 10 min at room temperature. Subsequently, 50 µl of LA solution was added to the mixture to start the reaction. After 10 min, solution A (270 µl) and then solution B (130 µl) were added to start color formation (preparation of solution A and B described in the previous section). 3 min later, 200 µl of a 2% SDS solution was added to terminate the reaction. The absorbance was recorded at 598 nm compared with the control. These experiments were performed in triplicate. Using the reaction rates (Abs.min^-1^), both inhibitor and LA concentrations (µM), Michaelis-Menten and Lineweaver-Burk plots were graphed in GraphPad Prism 6.0.

***Synthesis***

*1-Allyloxy-4-nitrobenzene (* ***2*** *)*

A mixture of 4-nitrophenol (**1**) (0.50 mol, 69.5 g), allyl bromide (0.55 mol, 66.0 g) and anhydrous potassium carbonate (0.50 mol, 70.0 g) in dry acetone (150 ml) was refluxed for 16 hr. After cooling, the mixture was diluted with water (250 ml) and extracted with ether (2×150 ml). The combined organic layers were washed with NaOH (10%, 2×150 ml) and dried over sodium sulfate. After removal of the solvent the residual oil of **2** was distilled under reduced pressure (74.4 g, 83% yield).

*2-Allyl-4-nitrophenol (* ***3*** *)*

An equal mixture of allyl ether **2** and diphenyl ether (35 g) was heated (210 ^°^C) for 5 hr under nitrogen atmosphere. After cooling, the mixture was dissolved in 200 ml of ethyl acetate and extracted with NaOH (10%, 3×70 ml). 

The combined aqueous solutions were acidified with concentrated hydrochloric acid and extracted with methyl *tert*-butyl ether (3×70 ml). The combined organic layers were dried over sodium sulfate and filtered. After evaporating the ether, the product was obtained as sticky solid, which was recrystallized from *n*-heptane to obtained pure yellow crystals of **3 **(23.0 g, 65% yield).

*2-Allyl-1-isopropoxy-4-nitrobenzene (* ***4*** *)*

3.4 g (1.89 mmol) of **3** and DBU 5.7 g (2.85 mmol) were dissolved in benzene (40 ml) and then 2-bromopropane 7.1 g (3.78 mmol) was added to them. The mixture was refluxed for 24 hr. After cooling to room temperature, the mixture was diluted with water (150 ml) and washed with NaOH (10%, 2×100 ml) and concentrated hydrochloric acid. The organic layer was dried with anhydrous sodium carbonate. 

*3-Allyl-4-isopropoxy benzene amine (* ***5*** *)*

A mixture of **4** (18.0 g, 0.082 mol) and SnCl_2_.2H_2_O (113.0 g, 0.50 mol) in absolute ethanol (160 ml) was refluxed under nitrogen for 20 min. Then, the mixture was poured into cold water (400 ml) and the pH adjusted to about 10 by adding sodium bicarbonate. 3-Allyl-4-isopropoxy benzene amine (**5**) was extracted using ethyl acetate (3×80 ml).

Extraction of 3-Allyl-4-isopropoxy benzene amine (**5**) was performed using ethyl acetate (3×80 ml). The ether layers was combined and dried over anhydrous sodium sulfate. After evaporating the organic solvent, the crud product (**5**) was purified by distillation under reduced pressure (12.3 g, 79% yield).

*General procedure for the preparation of* ***6a-f***

To stirred solution of potassium carbonate (5 g, 35 mmol; in 3 ml of water) and amine (**5**) solution (0.5 g, 2.7 mmol; in 5 ml of acetone), desired carbonyl chloride derivative (2.7 mmol) was added and the mixture was stirred for 30 min. 

After completion of the reaction (checked by TLC), the mixture was diluted with a little water and extracted with ethyl acetate (80×2 ml). The organic layers were dried with sodium carbonate and the crud product was obtained by evaporating the solvent. Purification was carried out by crystallization from ethanol.

*N-(3-allyl-4-isopropoxyphenyl) cyclopropane carboxamide (* ***6a*** *)*

Brownish solid, mp 78-79 ^°^C , yield: 75%,; ^1^HNMR (301 MHz, Chloroform-*d*): 0.72 (dt, *J*=4.4 Hz, 6H), 0.98 (d, *J*=4.2 Hz, 2H), 1.23 (d, *J*=6.1 Hz, 6H),1.36 (d, *J*=16.5 Hz, 1H), 3.26 (d, *J*=6.8 Hz,, 2H), 4.41 (d, *J*=6.1 Hz, 2H), 4.99 (m, 2H), 5.87 (dd, *J*=17.1, 10.1 Hz, 1H), 6.71 (d, *J*=8.8 Hz, 1H), 7.14 (s, 1H), 7.28 (s, 1H), 7.34 (s, 1H),^ 13^CNMR (75 MHz, Chloroform-*d*) δ7.69, 15.56, 22.21, 34.47, 70.61, 113.82, 115.64, 119.11, 122.08, 130.40, 131.05, 136.87, 152.20, 171.67.

*N-(3-allyl-4-isopropoxyphenyl) cyclobutane carboxamide (* ***6b*** *)*

White solid, mp: 81-83 ^°^C, yield: 80%; ^1^HNMR (301 MHz, Chloroform-*d*): δ 1.30 (d, *J* = 6.1 Hz, 6H), 1.75 (m, 4H), 1.92 (m, 2H), 2.45 (m, 1H), 3.22 (d, *J*= 6.8 Hz, 2H), 4.37 (m, 1H), 4.94 (m, 2H), 5.84 (m, 1H), 6.67 (m, 1H), 7.77 (s, 1H), 6.95(s, 1H), 7.05 (d, *J*= 8.6 Hz, 1H), ^13^CNMR (75 MHz, Chloroform-*d*) δ 22.20, 34.50, 70.57, 113.94, 115.63, 121.10, 124.08, 130.59, 130.73, 136.79, 152.56, 154.77.

*N-(3-allyl-4-isopropoxyphenyl) cyclopentane carboxamide (* ***6c*** *)*

White solid, mp: 88-89 ^°^C, yield: 85%; ^1^HNMR (301 MHz, Chloroform-*d*): δ 1.30 (d, *J*=6.1 Hz, 6H), 1.66 (m, 2H), 1.81 (d, *J*=4 Hz, 2H), 1.92 (d, *J*=7.7 Hz, 4H), 2.66 (m, 1H), 3.37 (d, *J*=6.8 Hz, 2H), 4.5 (dt, *J*=12.1, 6.1, 6.1 Hz, 1H), 5.07 (m, 2H), 5.98 (m, 1H), 6.82 (d, *J*= 8.6 Hz, 1H), 7.18 (s, 1H). 7.25(d, *J*=2.8 Hz, 1H), 7.41 (d, *J*= 11.4 Hz, 2H), ^13^CNMR (75 MHz, Chloroform-*d*) δ22.20, 26.04, 30.57, 34.49, 46.75, 70.62, 113.87, 115.64, 119.01, 121.99, 130.43, 131.06, 136.87, 152.18. 

*N-(3-allyl-4-isopropoxyphenyl) cyclohexane carboxamide (* ***6d*** *)*

White solid, mp 93-95 ^°^C, yield: 89%; ^1^HNMR (301 MHz, Chloroform-*d*): δ 1.33 (m, 8H), 1.57 (m, 2H), 1.70 (m, 2H), 1.85 (m, 2H), 1.97 (d, *J*=13.4 Hz, 2H), 2.22 (m, 1H), 3.37 (d, *J*=6.6 Hz, 2H), 4.51 (m, 1H), 5.07 (m, 2H), 5.98 (m, 1H), 6.83 (d, *J*=8.8 Hz,, 1H), 7.13 (s, 1H), 7.23 (d, *J*=2.6 Hz,, 1H), 7.41(s, 1H), ^13^CNMR (75 MHz, Chloroform-*d*) δ 22.19, 25.74, 29.74, 34.48, 46.45, 70.63, 113.87, 115.65, 119.08, 122.03, 130.44, 130.94, 136.85, 152.22.

*N-(3-allyl-4-isopropoxyphenyl) benzamide (* ***6e*** *)*

Yellowish solid, mp 128-130 ^°^C, yield: 89%; ^1^HNMR (301 MHz, Chloroform-*d*): δ 1.26 (d, *J*=6.1 Hz, 6H), 3.30 (d, *J*=6.8 Hz, 2H), 4.40 (m, 1H), 4.99 (m, 2H), 5.90 (m, 1H), 6.78 (d, *J*= 8.8 Hz, 1H), 7.23 (d, *J*=2.6 Hz, 1H), 7.41 (m, 4H), 7.69 (s, 1H), 7.78 (d, *J*=6.8 Hz, 2H), ^13^CNMR (75 MHz, Chloroform-*d*) δ 22.21, 34.48, 70.61, 113.79, 115.78, 119.63, 122.53, 126.99, 128.74, 130.54, 131.65, 135.15, 136.8, 152.64.

*N-(3-allyl-4-isopropoxyphenyl) adamantane carboxamide (* ***6f*** *)*

White solid, mp 188-1909 ^°^C, yield: 89%; ^1^HNMR (301 MHz, Chloroform-*d*): δ 1.34 (d, *J*=6.1 Hz, 6H), 1.78 (m, 6H), 1.98 (d, *J*=2.8 Hz, 6H), 2.12 (s, 3H), 3.38 (d, *J*=6.6 Hz, 2H), 4.51 (m, 1H), 5.08 (m, 2H), 5.98 (m, 1H), 6.84 (d, *J*=8.8 Hz, 1H), 7.21 (s, 1H), 7.25 (s, 1H), 7.42 (m, 1H), ^13^C NMR (75 MHz, Chloroform-*d*) δ 22.20, 29.75, 34.49, 38.21, 40.61, 45.70, 46.54, 70.73, 113.84, 115.72, 118.92, 122.12, 130.56, 130.98, 136.85, 152.25.

## Results

**Figure 1:**
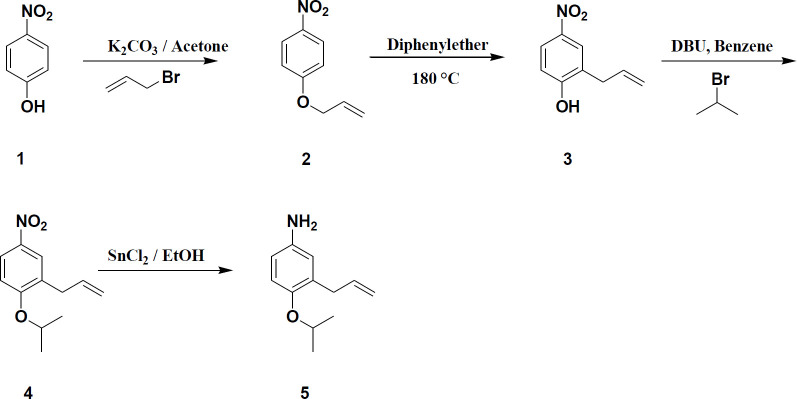
General procedure for preparation of 3-allyl-4-isopropoxy benzeneamine

**Figure 2:**
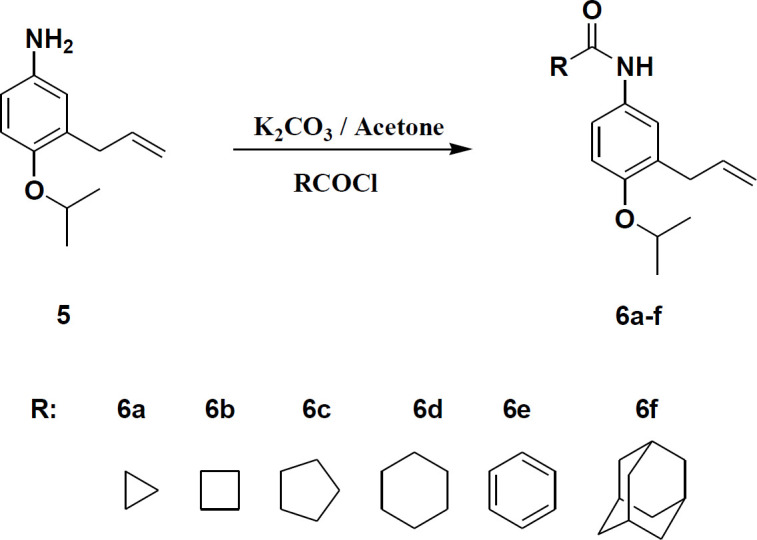
General procedure for synthesis of **6a-f**

Compound **5** as starting material was synthesized via four step reaction according to the procedure reported in a previous work (6, 10). The synthetic route for preparation of **5** is shown in Figure 1. Final products (**6a-f**) were prepared via amidation of **5** with the desired acyl chloride (Figure 2).

In the previous works on 2-Alkoxy-5-methoxyallylbenzenes, it was found that 2-isoproxy derivate (**A2**) was the most potent lipoxygenase inhibitor amongst their analogs (7). In the other works on N-(3-allyl-4-(allyloxy)phenyl) carboxamides, it was found that the size of aliphatic carboxamides had a predominant effect on lipoxygenase inhibition, so that N-(3-allyl-4-(allyloxy)phenyl) adamantanecarboxamide (**A1**) with the largest size showed the best inhibitory activity (9). Finally it was supposed that by replacing of *ortho* allyoxy moiety with isopropoxy in **A1** we could obtain a more potent inhibitor. Based on the mentioned hypothesis, N-(3-allyl-4-isopropoxyoxyphenyl) adamantanecarboxamide (**6f**) was synthesized. Like previous studies on the effect of carboxamide size on lipoxygenase inhibitory potency, similar cyclic aliphatic carboxamide derivatives of *ortho* isopropoxy allylbenzene were synthesized and their lipoxygenase inhibitory activity was determined (Table 1).

Lipoxygenase inhibitory activity of the synthetic compounds (substrate: linoleic acid) was assessed by using the peroxide-formation method, according to the published literature (14, 15). In this method, measurement of linoleate hydroperoxide concentration is the basis of lipoxygenase activity determination. All the assessments were performed in comparison with 4-MMPB (4-methyl-2-(4-methylpiperazinyl)pyrimido[4,5-b]benzothiazine) as a well-known lipoxygenase inhibitor. The enzyme inhibition results are mentioned in Table 2. 

**Figure 3:**
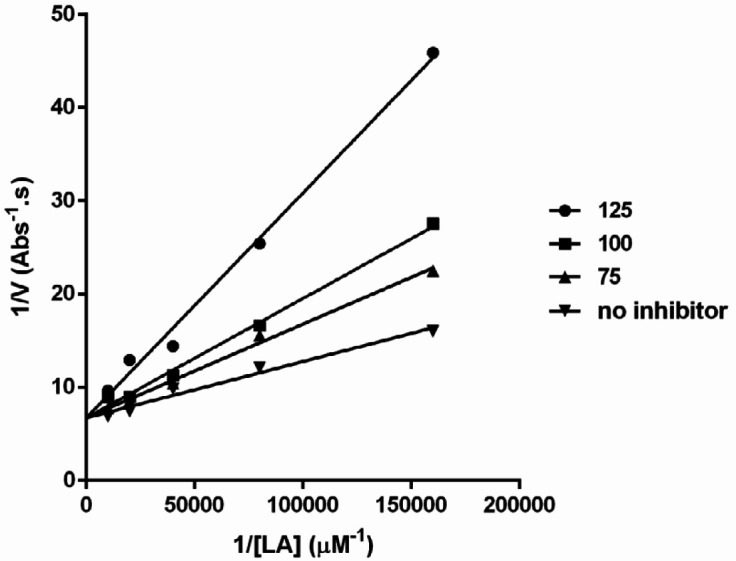
Lineweaver-Burk plot of SLO inhibition by** 6f **LA

SLO: soybean 15-LOX; LA: Linoleic acid

In this study, it was also determined that **6f** inhibited SLO by the competitive mechanism (Figure 3). The observed inhibitory mechanism showed that the synthetic compounds inhibited the enzyme activity without any direct interaction with iron core of SLO (iron chelation) or interfering with peroxidation cycle (Red-Ox mechanism). So molecular docking by AutoDockTools could be responsible for finding the probable binding conformation of the inhibitors in active site pocket of SLO. 

**Figure 4.**
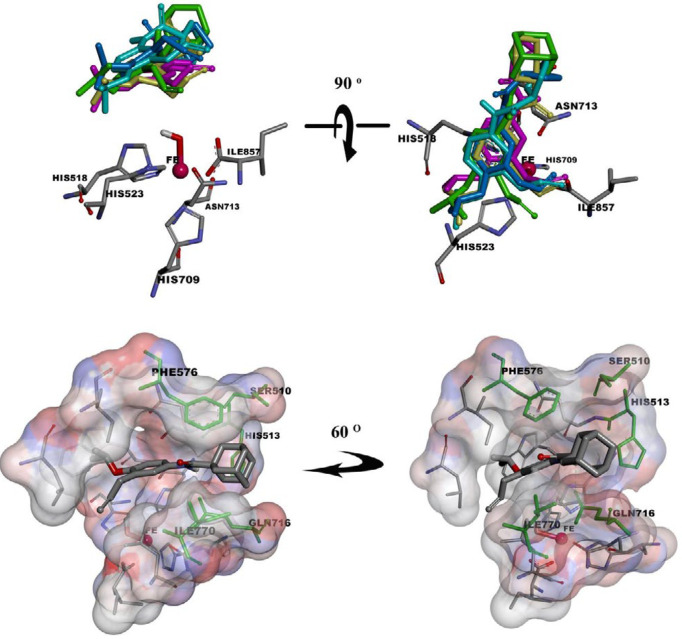
Orientation of consensus structures of **6a**, **6b**, **6c**, **6d**, and **6f** toward Fe^3+^-OH in SLO active site (above). Orientation of 6f in SLO active site pocket which is illustrated by solvent surface view (below)

**Figure 5:**
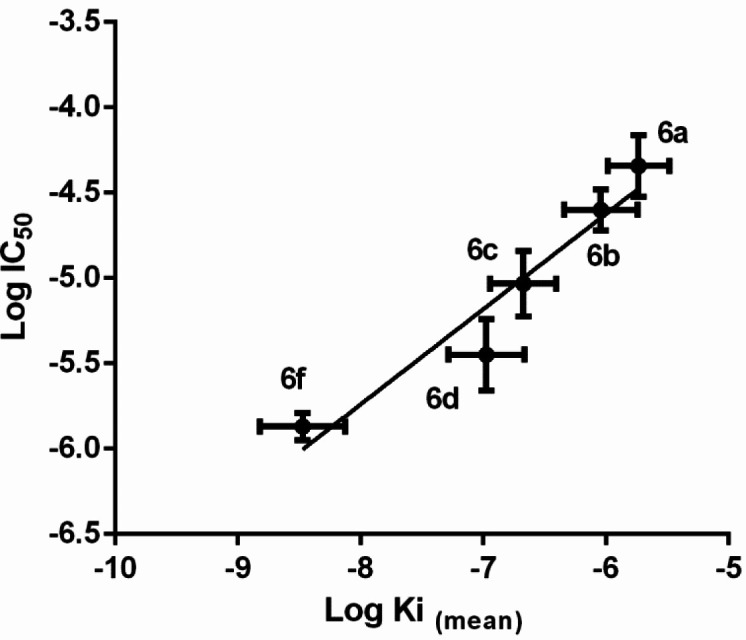
Diagram of log IC_50_ versus log Ki (mean) of compounds **6a–d **and **6f**

Geometrically minimized structures of the synthetic compounds, except **6e**, were docked into LOX (PDB entry: 1IK3). Free energy of binding (ΔG_b_) and inhibition constant (Ki) were calculated for each modelled binding conformer. Docking analysis on the resulted models by clustering with RMSD = 2Ǻ, outputted 10–15 clusters for each compound. It was interesting that the 1st to 3rd clusters, which had the largest average binding energies, were the most populated ones (12–23 docked conformers in a cluster) (Table 3) and also it was found that in the mentioned clusters the conformers had a similar orientation in which allyl moiety was directed toward iron core (Fe^3+^-OH) and their amide moieties were located in a cavity formed by Ser510, His513, Phe576 Gln716, and Ile770 (Figure 4). The desired docked models were fixed in the active site pocket by forming H-bonds between amide bond and Gln716 or His513 side chains. It was interesting that LOX inhibition potency variation of the synthetic compounds was in accordance with average Ki of clusters with the aforementioned description (Figure 5).

**Table 1:** Inhibitory potency of N-(3-allyl-4-(allyloxy)phenyl) adamantanecarboxamide (A1), 2-allyl-1-isopropoxy-4-methoxybenzene (A2) and **6f**

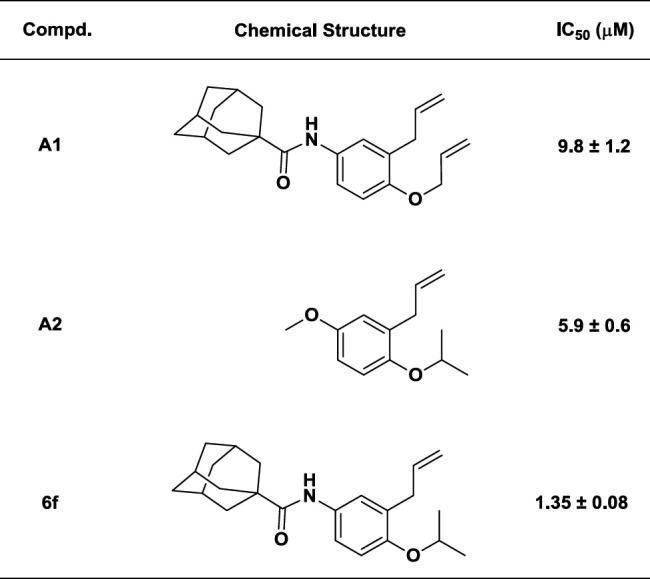

**Table 2:** The SLO inhibition results of the synthetic compounds in comparison with 4-MMPB (IC_50_= 9.8 µM) SLO: soybean 15-LOX

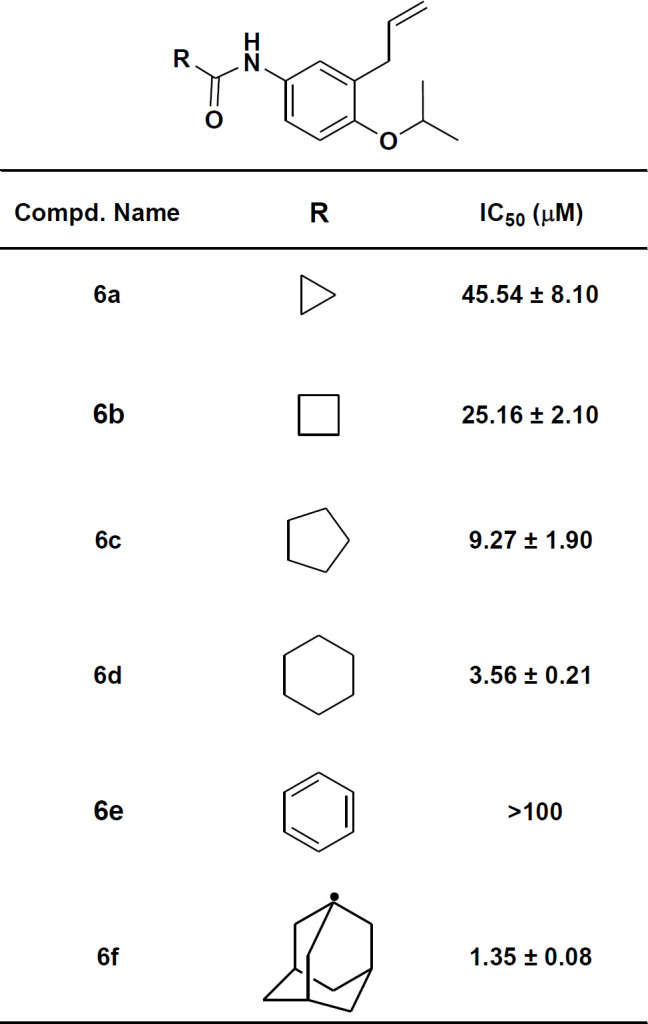

**Table 3:** Docking analysis results of **6a-f**. ΔGb: Estimated free energy of binding of the best conformer in cluster, ΔGb (mean): Average of estimated free energy of binding of all conformers in cluster, Ki (mean): estimated inhibition constant that has been calculated from ΔGb (mean)

Compd.	Cluster no.	Number of conformers in cluster	ΔG_b_ (Kcal/mol)	ΔG_b (mean)_ (Kcal/mol)	-Log Ki _(mean)_
**6a**	3	23	-8.47	-7.81 ± 1.4	5.735
**6b**	3	15	-9.03	-8.23 ± 1.6	6.043
**6c**	2	17	-9.86	-9.09 ± 1.5	6.674
**6d**	2	12	-10.45	-9.50 ± 1.7	6.976
**6f**	1	16	-12.69	-11.54 ± 1.9	8.473

SLO: soybean 15-LOX; LOX: Lipoxygenases

## Discussion

As mentioned in results section, it was predicted that by replacing of *ortho* allyoxy moiety with isopropoxy in **A1**, we could obtain a more potent inhibitor. According to the hypothesis, both insertion of isopropoxy group and increase of amid moiety size led to positive effects on inhibitory potency of the synthetic amides. **6f** showed the highest lipoxygenase inhibitory potency (IC_50_= 1.35±0.08 µM) while N-(3-allyl-4-isopropoxyphenyl) cyclopropane carboxamide (**6a**) was the weakest inhibitor (IC_50_=45.54±8.10 µM). Compound **6e** did not show any activity, so it was omitted from docking analysis. л electron interaction of benzene portion of the amide moiety with amino acid side chains (л^*^- л and ẟ^*^- л interactions), possibly could lead to different direction of **6e** in the SLO active site pocket and subsequently loss of its inhibitory activity. As AutoDockTools could not calculate such interactions, docking analysis would not be trustable.

As mentioned in the results section, LOX inhibition potency variation of the synthetic compounds (IC_50_) was in accordance with average Ki of clusters comprising conformers with the specific orientation in SLO active site pocket (Figure 5). In this orientation, allyl moiety is directed toward iron core (Fe^3+^-OH), and the amide moieties are located in a cavity formed by Ser510, His513, Phe576 Gln716, and Ile770 (Figure 4). The interior part of the cavity has considerable lipophilic properties and makes a suitable space for location of aliphatic portion of the amide moiety. H-bonds between the amide bond and Gln716 or His513 side chains would fix the mentioned orientation in the active site pocket.

## Conclusion

By chemical structure modification on allylbenzene substituents we could obtain the more potent LOX inhibitors. Docking results showed that orientation of the amide and allyl moieties of the inhibitors in the active site pocket of SLO plays a basic role in inhibitory potency variation. Based on the mentioned orientation, for cycloaliphatic amides, by enlargement of the amide moiety both inhibitory potency and calculated binding energy increases. Finally, these findings provide a structure template for synthesis of new compounds with larger and branched cyclo aliphatic amides to obtain more potent LOX inhibitors.

## References

[1] Kuhn H, Borngraber S (1999). Mammalian 15-lipoxygenases Enzymatic properties and biological implications. Adv Exp Med Biol.

[2] His LC, Wilson LC, Eling TE (2002). Opposing effects of 15-lipoxygenase-1 and-2 metabolites on MAPK signaling in prostate Alteration in peroxisome proliferator-activated receptor gamma. J Biol Chem.

[3] KuÈhn H, Barnett J, Grunberger D, Beacker P, Chow J, Nguyen B (1993). Overexpression, purification and characterization of human recombinant 15-lipoxygenase. J Biochim Biophys Acta.

[4] KuÈhn H, Thiele BJ, Ostareck-Lederer A, Stender H, Suzuki H, Yoshimoto T (1993). Bacterial expression, purification and partial characterization of recombinant rabbit reticulocyte 15-lipoxygenase. J Biochim Biophys Acta.

[5] Alavi SJ, Sadeghian H, Seyedi SM, Salimi A, Eshghi H (2018). A novel class of human 15-LOX-1 inhibitors based on 3-hydroxycoumarin. Chem Bio Drug Des.

[6] Jabbari A, Davood Nejad M, Alimardani M, Assadieskandar A, Sadeghian A, Safradi H (2012). Synthesis and SAR studies of 3-allyl-4-prenyloxyaniline amides as potent 15-lipoxygenase inhibitors. J Bioorg Med Chem.

[7] Sadeghian H, Jabbari A (2016). 15-Lipoxygenase inhibitors: a patent review. Expert Opin Ther Pat.

[8] Van Leyen K, Kim HY, Lee SR, Jin G, Arai K, Lo EH (2006). Biacalein and 12/15- lipoxygenase in the ischemic brain. Stroke.

[9] Mytilineou C, Kramer BC, Yabut JA (2002). Glutathion depletion and oxidative stress. Parkinsonism. Relat. Disord..

[10] Sadeghian H, Seyedi M, Attaran N, Jabbari A, Jafari Z (2011). Synthesis and SAR comparative studies of 2-allyl4-methoxy-1-alkoxybenzenes as 15-lipoxygenase inhibitors. J Enzyme Inhib Med Chem.

[11] Seyedi M, Jafari Z, Attaran N, Sadeghian H, Saberi MR, Riazi MM (2009). Design, synthesis and SAR studies of 4-allyoxyaniline amides as potent 15-lipoxygensae inhibitors. Bioorg Med Chem.

[12] Sadeghian H, Seyedi SM, Saberi MR, Arghiani Z, Riazi M (2008). Design and synthesis eugenol derivatives, as potent 15-lipoxygenase inhibitors. Bioorg Med Chem.

[13] Sadeghian H, Attaran N, Jafari Z, Saberi MR, Seyedi, SM, Eshghi H (2009). Design and synthesis of 4-methoxyphenylacetic acid esters as 15-lipoxygenase inhibitors and SAR comparative studies of them. Bioorg Med Chem.

[14] Iranshahi M, Jabbari A, Orafaie A, Mehri R, Zeraatkar S, Ahmadi T (2012). Synthesis and SAR studies of mono O-prenylated coumarins as potent 15-lipoxygenase inhibitors. Eur J Med Chem.

[15] Nikpour M, Mousavian M, Davoodnejad M, Alimardani M, Sadeghian H (2013). Synthesis of new series of pyrimido[4,5-b][1,4] benzothiazines as 15-lipoxygenase inhibitors and study of their inhibitory mechanism. Med Chem Res.

